# Effectiveness of Seizure Dogs for People With Severe Refractory Epilepsy: Results From the EPISODE Study

**DOI:** 10.1212/WNL.0000000000209178

**Published:** 2024-02-28

**Authors:** Valérie van Hezik-Wester, Saskia de Groot, Tim Kanters, Louis Wagner, Jacqueline Ardesch, Werner Brouwer, Isaac Corro Ramos, Saskia le Cessie, Matthijs Versteegh, Job van Exel, for the EPISODE team

**Affiliations:** From the Erasmus School of Health Policy & Management (ESHPM) (V.H.-W., S.G., T.K., W.B., I.C.R., M.V., J.E.), Institute for Medical Technology Assessment (iMTA) (V.H.-W., S.G., T.K., I.C.R., M.V.), and Erasmus Centre for Health Economics Rotterdam (EsCHER) (V.H.-W., S.G., T.K., W.B., I.C.R., M.V., J.E.), Erasmus University Rotterdam; Academic Center for Epileptology Kempenhaeghe (L.W.), Heeze; Stichting Epilepsie Instellingen Nederland (SEIN) (J.A.), Heemstede; Leiden University Medical Center (S.C.); Huygens & Versteegh (M.V.), Zwijndrecht, the Netherlands.

## Abstract

**Background and Objectives:**

The aim of this study was to evaluate whether people living with severe medically refractory epilepsy (PSRE) benefit from a seizure dog.

**Methods:**

An individual-level stepped-wedge randomized controlled trial was conducted. The study was conducted in the Netherlands among adults with daily to weekly seizures. All participants were included simultaneously (on June 1, 2019) while receiving usual care. Then, during the 36-month follow-up, they received a seizure dog in a randomized sequence. Participants kept a seizure diary and completed 3-monthly surveys. Seizure frequency was the primary outcome. Secondary outcomes included seizure-free days, seizure severity, health-related quality of life (HRQoL), and well-being. Data were analyzed using generalized linear mixed modeling (GLMM). The models assumed a delayed intervention effect, starting when the seizure dog reached an advanced stage of training. Effects were calculated as changes per 28-day period with the intervention.

**Results:**

Data were collected from 25 participants, of whom 20 crossed over to the intervention condition. The median follow-up was 19 months with usual care and 12 months with the intervention. On average, participants experienced 115 (SD 164) seizures per 28-day period in the usual care condition and 73 (SD 131) seizures in the intervention condition. Seven participants achieved a reduction of 50% or more at the end of follow-up. GLMM indicated a 3.1% decrease in seizure frequency for each consecutive 28-day period with the intervention (0.969, 95% CI 0.960–0.977). Furthermore, an increase in the number of seizure-free days was observed (1.012, 95% CI 1.009, 1.015), but no effect on seizure severity measured with the NHS3. Generic HRQoL scores improved, as reflected in the decrease in EQ-5D-5L utility decrement (0.975, 95% CI 0.954–0.997). Smaller improvements were observed on overall self-rated HRQoL, epilepsy-specific HRQoL, and well-being, measured with the EQ VAS, QOLIE-31-P, and ICECAP-A, respectively.

**Discussion:**

Seizure dogs reduce seizure frequency, increase the number of seizure-free days, and improve the quality of life of PSRE. The magnitude of the effect on generic HRQoL indicates that seizure dogs benefit PSRE beyond the impact on seizure frequency alone. Early discontinuation of seizure dog partnerships suggests that this intervention is not suitable for all PSRE and requires further study.

**Trial Registration Information:**

This study was registered in the Dutch Trial Register (NL6682) on November 28, 2017. Participants were enrolled on June 1, 2019.

**Classification of Evidence:**

This study provides Class III evidence that seizure dogs are associated with a decrease in seizure frequency in adult patients with medically refractory epilepsy.

## Introduction

Epilepsy imposes a significant clinical and economic burden on societies. Despite the development of numerous antiseizure medications over the past 15 years, approximately 20%–30% of people with epilepsy experiences persistent seizures.^1^ While epilepsy surgery can be effective in eliminating seizures, only a small minority of people with epilepsy is eligible for surgery.^2^ Neurostimulation is another treatment alternative but does not often result in seizure freedom.^3^ Hence, a proportion of people with epilepsy experiences frequent seizures despite the wide and continuously expanding range of treatments. People living with severe medically refractory epilepsy (PSRE) bear the greatest burden of epilepsy-related disabilities and are at risk of falls, drowning, and burn wounds.^4^ Furthermore, depression and anxiety disorders are important comorbid conditions in those who experience frequent seizures.^5,6^

The unpredictable nature of seizures is generally considered the most disabling aspect of the condition.^7,8^ Many seizures are accompanied by loss of consciousness, and PSRE are often unable to call for help. Timely intervention on the occurrence of a seizure, such as administering emergency medication, can reduce the risk of seizure-related injuries, status epilepticus, and sudden unexpected death. Therefore, over the past few years, wearable devices have been developed to detect seizures and alert caregivers.^9^ Yet, no device is currently able to recognize all types of seizures due to their different clinical manifestations. Moreover, the risk of false positives resulting from everyday activities restricts the usability of most devices to nighttime.

Seizure dogs may help overcome some of the limitations of seizure detection devices. These formally trained dogs recognize seizures and respond when they occur. They are trained to identify seizures activity in the person they are partnered with by observing body movements, sounds, and physiological signals. The set of response tasks depends on the care needs of the person with epilepsy, but generally includes the activation of an alarm system, fetching medication or a phone, blocking the person's movement, or changing the person's body position. Furthermore, the dog can provide companionship as the seizure subsides, a period during which the person may feel disoriented and anxious. Previous exploratory studies suggested that seizure dogs could potentially reduce seizure frequency and improve quality of life.^10-12^ Stress is the most common trigger for seizures, with half of people with epilepsy reporting stress-precipitated seizures.^8,13-15^ The tasks seizure dogs perform and their companionship may alleviate (seizure-related) anxiety, potentially reducing stress-precipitated seizures. Furthermore, seizure dogs may facilitate rapid action when a seizure occurs, limiting the risk of seizure clusters and seizure-related injuries. However, current evidence for the benefits of seizure dogs is limited, which hinders their consideration as routine (reimbursed) care.^16,17^ At the same time, because it concerns a costly intervention that not many people with epilepsy can afford, the current number of seizure dogs is very low and (opportunities for collecting) observational data thus also limited.

The primary aim of the EPISODE (EPIlepsy SuppOrt Dog Evaluation) study was to evaluate whether seizure frequency is reduced by the provision of a seizure dog in addition to usual care, relative to usual care alone, in adults with severe medically refractory epilepsy.^18^ Because previous studies suggest that seizure dogs may affect the lives of PSRE more broadly, the secondary aim was to evaluate the impact of seizure dogs on seizure-free days, seizure severity, health-related quality of life (HRQoL), and well-being.

## Methods

The methods of this study are described further. A detailed description of the EPISODE study rationale and methods can be found in the published study protocol.^18^

### Study Design

An individual-level stepped-wedge design was adopted. This is a subtype of randomized controlled trials (RCTs) in which the intervention is gradually introduced to the study population. Randomization determines the point in time during which participants receive the intervention, rather than whether or not they receive the intervention at all as in traditional RCTs. The study was conducted in the Netherlands. Participants were enrolled on June 1, 2019, and followed up for 3 years, until May 31, 2022. Participants were first observed for a baseline period (i.e., usual care condition), after which they sequentially received a seizure dog at their assigned time slot and were transferred to the intervention condition in a randomized sequence.

### Eligibility Criteria and Screening Process

People were eligible for study participation if they had medically refractory epilepsy, an average seizure frequency of 2 per week or more, seizure characteristics associated with a high risk of injuries or dysfunction, and the ability to care for a seizure dog (full set of criteria available in eAppendix 1, eTable 1, links.lww.com/WNL/D441). Eligibility was assessed by the treating neurologist and had to be confirmed by a neurologist in the study team. In addition, the seizure dog provider advised on the feasibility of starting a seizure dog trajectory considering the applicant's personal circumstances (e.g., housing conditions and support network to help with dog care and training).

### Intervention Characteristics

The intervention was defined as the partnership with a dog that is being trained or has finished a training trajectory focused on recognizing seizures and responding when they occur. Seizure dogs may also develop alerting behavior, which consists of anticipating on an impending seizure.^17^ Seizure dog trainers were attentive to signs of such behavior, but the cues that allow some dogs to anticipate seizures are unknown and, therefore, alerting behavior cannot be trained. Seizure dogs were provided through either a pretrained dog trajectory or a team coaching trajectory. In the pretrained dog trajectory, the participant was partnered with a dog that had finished socialization and obedience training, after which the training of epilepsy-specific tasks was continued at the participant's home. In the team coaching trajectory, participants were coached in training a puppy in their own home from the start. Participants were allocated to the trajectory of their preference. Because both trajectories aimed to provide a seizure dog that adheres to the standards of Assistance Dogs International,^19^ the effect of the trajectories was assumed to be identical. Usual care included treatments to control seizures, such as antiseizure medications and neurostimulation, and assistive care services and technologies, such as occupational therapy and wearable alarm devices.

### Randomization

Before the start of data collection, participants were randomly assigned to a time point at which their seizure dog trajectory would start. The randomization was conducted separately for the pretrained dog trajectory and the team coaching trajectory, taking into account the seizure dog providers' capacities and a minimum follow-up of 3 months without a study dog and 3 months with a certified seizure dog for each participant. As a good fit with the dog was considered a crucial factor for the success of a seizure dog partnership, deviation from the randomized order was allowed when there was no match between the participant and the dog(s) available at the assigned time point.

### Stepped-Wedge Design Specification

**Figure 1:**
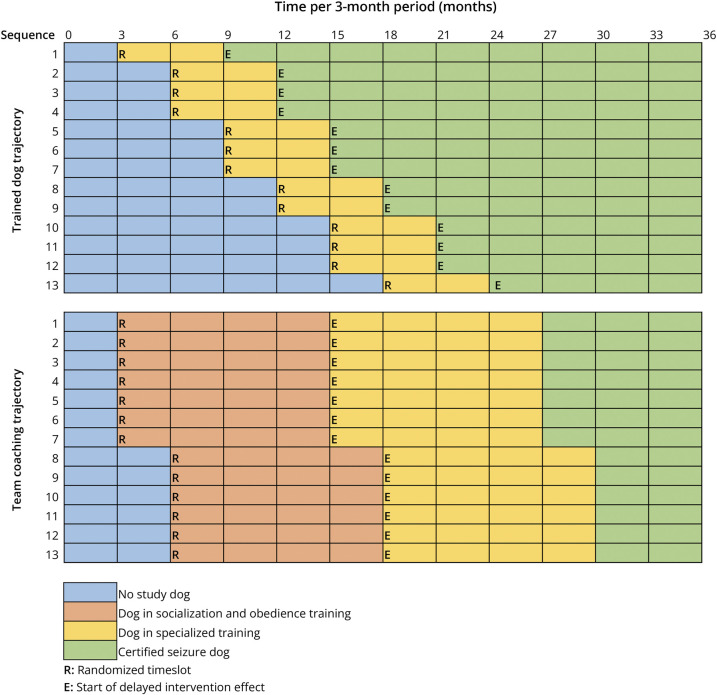
Stepped-Wedge Schedule Reflecting the Planned Rollout and Different Stages of Seizure Dog Trajectories Note: adapted from Wester V, de Groot S, Kanters T et al. Evaluating the Effectiveness and Cost-Effectiveness of Seizure Dogs in Persons With Medically Refractory Epilepsy in the Netherlands: Study Protocol for a Stepped-Wedge Randomized Controlled Trial (EPISODE). Front Neurol. 2020;11:3. Each row reflects 1 participant. Based on the inclusion during randomization, the schedule was designed to randomize 26 participants. They were equally divided over the 2 seizure dog trajectories. The columns reflect time in 3-month periods, totaling to the 3-year follow-up. As time progressed, more participants were scheduled to have switched from the usual care condition to the intervention condition.

Figure 1 presents the stepped-wedge schedule reflecting the individual pathways to which participants were randomized, stratified by seizure dog trajectory. The crossover between usual care and the intervention conditions was defined as 6 months after placement in the pretrained dog trajectory and as the seizure dog passing the socialization and obedience test in the team coaching trajectory (approximately 12 months after placement of the puppy). This cutoff was defined before data collection and taken into account in the statistical analyses. It was based on the hypothesis that the dog starts providing seizure dog–specific benefits when the participant and the dog have bonded, and training is focused on epilepsy-specific tasks.^18^

### Data Collection

Seizure frequency was the primary outcome of the study. Using paper seizure diaries, participants recorded their daily seizure counts for up to 3 most frequently occurring and countable seizure types. Each week, participants submitted a photograph of their seizure diary through a smartphone application. For the analysis, daily seizure counts were converted to obtain cumulative seizure frequencies over 28-day periods. Participants completed a survey every 3 months. The survey consisted of a set of validated questionnaires, including the NHS3^20^ to measure seizure severity, the EQ-5D-5L,^21^ EQ VAS,^21^ and QOLIE-31-P^22^ to measure HRQoL, and the ICECAP-A^23^ to measure well-being.

### Sample Size Calculation

In an earlier observational study on seizure alert dogs, an average decrease of 43% in 28-day seizure frequency was observed in 10 individuals 24–36 weeks after pairing with a seizure dog.^9^ To determine statistical power for this study, 2,500 simulations were run incorporating the planned analyses and stepped-wedge schedule. The power was calculated as the proportion of simulations that detected the intervention effect at a 5% significance level. Two sample sizes were tested: one with 20 participants and another with 25 participants. With both sample sizes, the study would have more than 80% power to identify a reduction in seizure frequency similar to the effect previously demonstrated.^18^

### Handling Missing Data

When information on an outcome measure was missing in full (i.e., unit nonresponse), no imputation was conducted. When information was missing partially (i.e., item nonresponse), missing values were imputed to retain observations in the dataset. For 28-day seizure frequency, a missing daily seizure count was imputed with the participant’s mean seizure count in the particular period. An exception was made when a participant noted the seizure count was missing because of the unusual high frequency, for example, due to clustering or a status epilepticus. In those cases, the missing daily seizure count was imputed with the highest seizure count recorded by the participant over the entire follow-up. For the NHS3, EQ-5D-5L, and ICECAP-A, missing item scores were imputed with the mean of the participant’s nearest nonmissing prior and posterior observations for that item.^24^ For the QOLIE-31-P, the scoring manual was followed for handling missing data.^22^

### Statistical Analysis

Data were analyzed in accordance with a prespecified statistical analysis plan.^18^ To account for repeated measures of participants over time, the effects of seizure dogs were examined using a generalized linear mixed modeling (GLMM) approach. For all outcomes, effects were assumed to develop linearly over time with the intervention. Time was expressed in 28-day periods, and consequently, effects were reported as changes per 28-day period with the intervention. Specifically, the GLMM analyses included a parameter for time with the intervention as a fixed effect and a random effect for each participant.

For the primary outcome, the statistical analysis plan prescribed a GLMM approach with a Poisson distribution with a log link. The observed seizure frequency data exhibited an unexpected high degree of overdispersion, which may result in biased parameter estimates and invalid conclusions when using this distribution.^25^ Therefore, an observation-level random effect was added where each data point receives its own random effect.^25^ To test the robustness of the results for model specifications and assumptions, sensitivity analyses were performed. These analyses included the exclusion of absence and myoclonic seizures and different approaches to account for the effect of time.

For secondary outcomes, an appropriate distribution family and link function were chosen depending on the observed distribution of the dependent variable. For the EQ-5D-5L and ICECAP-A, utility scores were calculated using tariffs for the Netherlands.^26,27^ Utility decrements (=1 minus the utility score) were used in the effect estimations for the EQ-5D-5L. Details of all models are presented in eAppendix 1, eTable 2 (links.lww.com/WNL/D441). Data analysis was performed in R software.

### Standard Protocol Approvals, Registrations, and Patient Consent

This study was registered with the Dutch Trial Register (NL6682) on November 28, 2017. Participants were enrolled in the study on June 1, 2019. All participants provided written informed consent. The Medical Ethics Committee of Erasmus Medical Center Rotterdam declared that the rules of the Medical Research Involving Human Subject Act do not apply to this study (MEC-2017-538). The study was approved by the Medical Ethics Committee Kempenhaeghe (METC 18.06).

### Data Availability

The individual patient data used in this article are not publicly available because approval was not obtained from participants to share their data publicly. Requests to access the data should be directed to the corresponding author.

## Results

### Inclusion and Rollout of the Intervention

**Figure 2:**
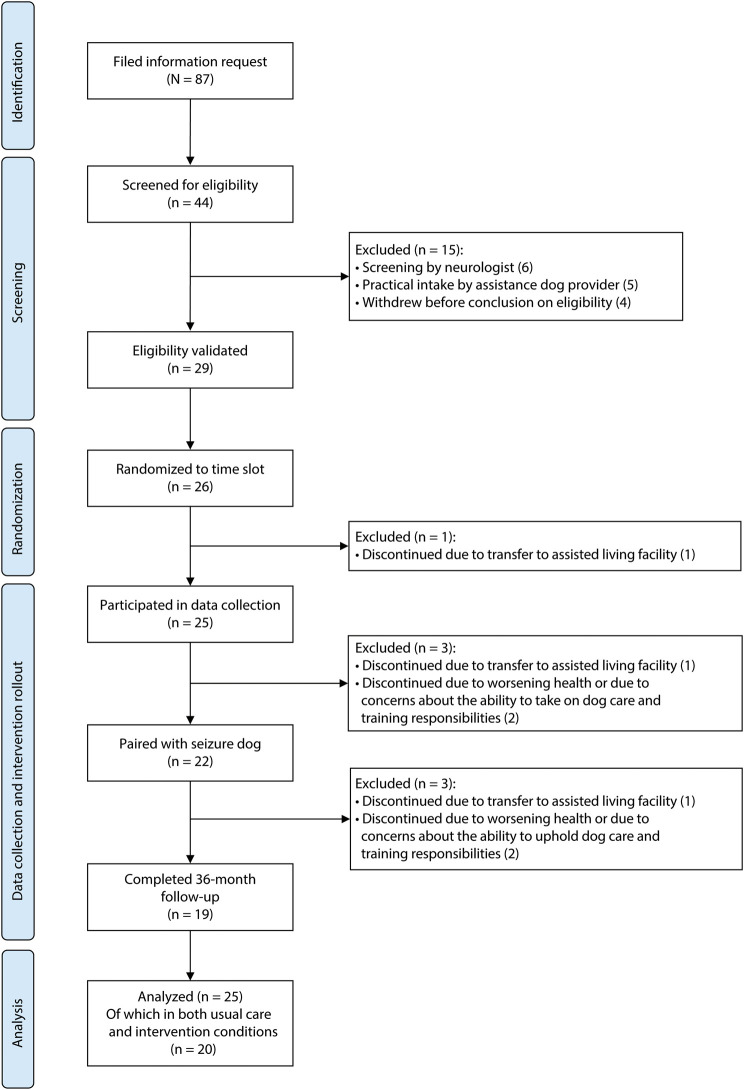
Trial Flow Diagram of Screening, Randomization, and Follow-up

Twenty-five PSRE participated in the study. Table 1 presents the characteristics of the participants at the start of the study. The trial flow diagram is presented in Figure 2. Six participants discontinued their study participation before the end of the trial follow-up. Of them, 3 participants discontinued before placement of the seizure dog. Three additional participants discontinued after placement, 2 of whom discontinued before the assumed start of the intervention effect and 1 thereafter. Consequently, 20 participants were observed under both the usual care and intervention conditions. eFigure 1 in eAppendix 1 (links.lww.com/WNL/D441) presents the final stepped-wedge schedule. Data on seizure frequency were available for 99% of the observed 28-day periods (846 of 851), and 95% of the surveys were returned (270 of 283). More information on missing data for each outcome measure is included in eAppendix 1, eTable 3.

**Table 1:** Sociodemographic and Clinical Characteristics at the Start of the Study (n = 25)

Characteristics	N (%)
Sociodemographic characteristics	
Sex	
Male	14 (56.0)
Female	11 (44.0)
Age (mean, SD, range)	33.8 (± 12.3, range 20–57)
Living situation	
Alone	2 (8.0)
With parents	12 (48.0)
With partner and/or children	11 (44.0)
Dog owner (before start of the study)	
Yes	8 (32.0)
No	17 (68.0)
Clinical characteristics	
Duration of disease in years (mean, SD)	22.6 (±14.1)
Type of epilepsy	
Focal onset	16 (64.0)
Generalized onset	7 (28.0)
Unknown onset	2 (8.0)
Number of seizure types recorded in seizure diary^a^	
1	3 (12.0)
2	12 (48.0)
3	10 (40.0)
Number of participants recording seizure type	
Focal-onset tonic-clonic seizure	13 (52.0)
Generalized-onset tonic-clonic seizure	6 (24.0)
Unknown-onset tonic-clonic seizure	1 (4.0)
Focal motor seizure impaired awareness	10 (40.0)
Focal nonmotor seizure impaired awareness	8 (32.0)
Focal motor seizure aware	1 (4.0)
Focal nonmotor seizure aware	1 (4.0)
Generalized motor seizure	6 (24.0)
Generalized nonmotor seizure (absence)	5 (20.0)
Not classifiable/unknown	6 (24.0)
Frequency of seizures during the first 28-day period^a^	
Daily	9 (36.0)
Three to 6 times a week	9 (36.0)
Twice a week or less	7 (28.0)
Seizure frequency on a seizure day during first 28-day period^a^ (median, range)	4 (1–29)
Comorbidity at baseline	
No comorbid conditions	10 (40.0)
1 comorbid condition	2 (8.0)
2–3 comorbid conditions	9 (36.0)
4 or more comorbid conditions	3 (12.0)
Missing	1 (4.0)

a Seizure types for which the participant could not record daily frequencies (e.g., because the seizures are difficult to notice or occur at a high frequency) are not considered.

### Seizure Frequency, Seizure-Free Days, and Seizure Severity

The median follow-up consisted of twenty-one 28-day periods in the usual care condition (range 3–36) and thirteen 28-day periods in the intervention condition (range 0–27), with a total follow-up ranging from three to thirty-nine 28-day periods (median 39). The number of observations for seizure frequency over time with the intervention can be found in eAppendix 1, eFigure 2 (links.lww.com/WNL/D441).

**Figure 3:**
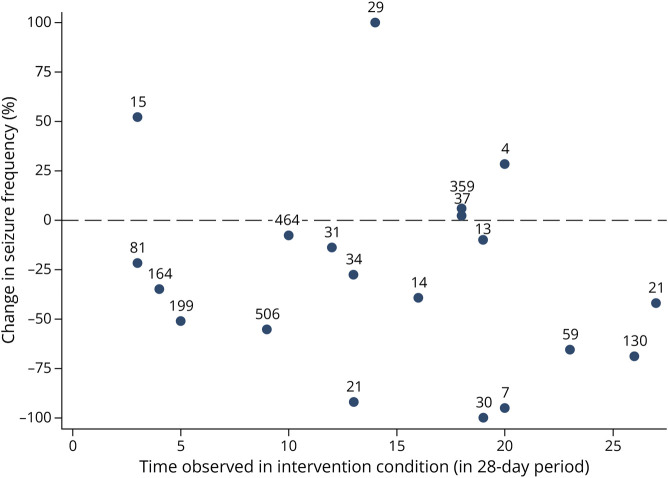
Change in Seizure Frequency as Percentage Change Over the Last Three 28-Day Periods in the Intervention Condition Relative to the Average Over the Total Time in the Usual Care Condition Each dot reflects 1 participant. Only participants observed with the intervention are presented. The number above each dot reflects the participant's average seizure frequency in the usual care condition. The x-axis reflects the time the participant is observed in the intervention condition.

Participants experienced an average of 115 (SD 164) seizures per 28-day period in the usual care condition and 73 (SD 131) seizures per 28-day period in the intervention condition (difference of −36.5%). The median seizure frequencies were 37.5 and 24, respectively (difference of −36.0%). The average seizure frequency over the last three 28-day periods (i.e., 12 weeks) of follow-up in the intervention condition was 31.1% lower when compared with the average seizure frequency in the usual care condition. A 25%–49% reduction in seizure frequency was observed in 4 participants, and a 50%–100% reduction in 7 participants (Figure 3). One participant had a 25%–50% increase in seizure frequency, and 2 participants showed an increase of 50% or more. For the remaining 6 participants, the change in seizure frequency was less than 25% (with 4 participants reporting a decrease and 2 an increase).

**Figure 4:**
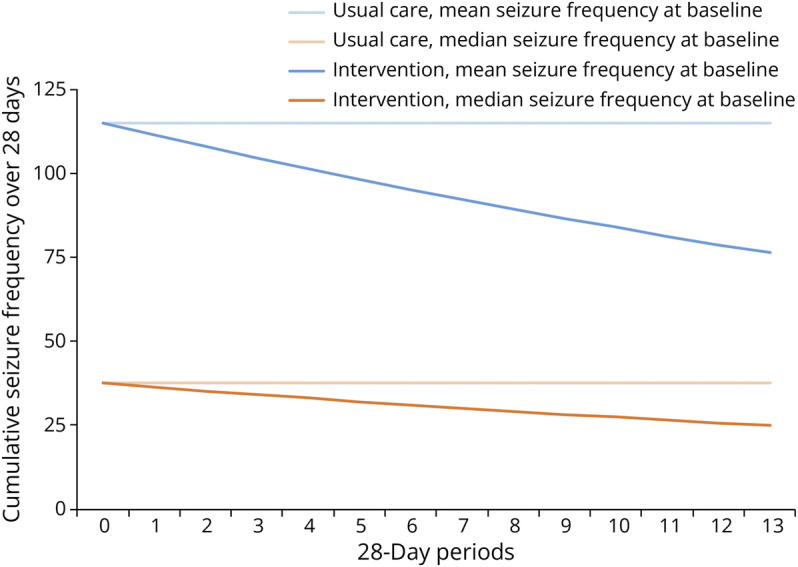
Estimated Effect Plotted Over 1 Year, Comparison Between Usual Care and Intervention Arm Using Mean and Median Seizure Frequency at Baseline as a Reference

For each consecutive 28-day period with the intervention, seizure frequency decreased on average by 3.1% (0.969, 95% CI 0.960–0.977) (Table 2). Figure 4 presents the estimated change over 1 year, using the mean and median seizure frequency of the study population at baseline as a reference.

**Table 2:** Study Results on Seizure Frequency, Seizure-free Day Count, Seizure Severity, HRQoL, and Well-Being: Outcomes of the Generalized Linear Mixed Models

	28-d seizure count	28-d seizure-free day count	NHS3 score	EQ-5D-5L utility score decrement	EQ VAS score	QOLIE-31-P score	ICECAP-A utility score
Regression results							
Exponentiated coefficient for time with the intervention	0.969	1.012	1.001	0.975	1.001	1.002	1.004
95% CI	0.960–0.977	1.009–1.015	1.000–1.002	0.954–0.997	1.001–1.002	1.001–1.002	1.001–1.006

Sensitivity analyses on 28-day seizure frequency showed effects in the same direction (eAppendix 1, eTable 4, links.lww.com/WNL/D441). Excluding absences and myoclonic seizures resulted in an average reduction of 3.4% for each consecutive 28-day period (0.966, 95% CI 0.957–0.974).

In the usual care condition, participants reported on average 11 (SD 9.8) seizure-free days per 28-day period, while in the intervention condition, this was 15 (SD 9.6). The number of seizure-free days increased on average by 1.2% for each consecutive 28-day period with the intervention (1.012, 95% CI 1.009–1.015) (Table 2). The intervention duration did not affect seizure severity as measured with the NHS3 (1.001, 95% CI 1.000–1.002).

### HRQoL and Well-Being

Participants completed the surveys a median of 7 times in the usual care condition (range 1–12) and 3 times in the intervention condition (range 0–9), with a total ranging from 1 to 13 completed surveys per participant (median 13).

The average utility score in the usual care condition was 0.674 (SD 0.262) on the EQ-5D-5L. In the intervention condition, the average score was 0.748 (SD 0.214). EQ-5D-5L utility decrements decreased on average by 2.5% per consecutive 28-day period with the intervention, reflecting an increase in generic HRQoL (0.975, 95% CI 0.954–0.997).

The average scores on the EQ VAS and QOLIE-31-P were 69.0 (SD 19.4) and 55.4 (SD 15.8), respectively, in the usual care condition, and 73.9 (SD 16.9) and 58.7 (SD 13.9), respectively, in the intervention condition. Therefore, for each consecutive 28-day period with the intervention, the EQ VAS score increased by 0.1% (1.001, 95% CI 1.001–1.002), reflecting an increase in the overall self-rated HRQoL. The QOLIE-31-P score increased by 0.2% each period (1.002, 95% CI 1.001–1.002), reflecting an increase in epilepsy-specific HRQoL. eFigure 3 in eAppendix 1 (links.lww.com/WNL/D441) provides a graphical representation of the estimated changes over 1 year for each QoL instrument.

In the usual care condition, the average ICECAP-A utility score was 0.738 (SD 0.187). In the intervention condition, the average score was 0.781(SD 0.164). ICECAP-A utility scores increased on average by 0.4% per consecutive 28-day period with the intervention (1.004, 95% CI 1.001–1.006), reflecting an increase in well-being.

Dimension score analyses indicated improvements over time with the intervention on the anxiety/depression dimension of the EQ-5D-5L and on the stability and achievement dimensions of the ICECAP-A. Moreover, improvements were observed on 5 of 7 dimensions of the QOLIE-31-P, with largest improvements on the social function and seizure worry dimensions. Scores on the other 2 dimensions worsened (cognition and medication side-effects). More detailed results on dimension scores are presented in eAppendix 1, eTable 5 (links.lww.com/WNL/D441).

## Discussion

The EPISODE study evaluated the effectiveness of seizure dogs using a randomized design. The intervention was targeted at a difficult-to-treat population, for whom currently no further treatment options exist. This population bears a substantial burden of illness, leaving a high unmet need for care.^28^ The study showed that partnering of PSRE with a seizure dog reduced seizure frequency, increased the number of seizure-free days, and improved quality of life.

Seizure frequency decreased by a rounded 3.1% each consecutive 28-day period with the intervention, resulting in a cumulative reduction of 33.9% after 1 year (i.e., 13 periods). This effect remained consistent across various modeling assumptions, consistently showing a decrease in seizure frequency with time with the intervention. Previously, only 1 study evaluated the effectiveness of trained seizure dogs. In that study, baseline seizure frequency was compared with the seizure frequency in the last 12 weeks of follow-up with the seizure alert dog. Four of 10 PSRE achieved a 50% reduction or more in tonic-clonic seizures.^10^ Considering all seizure types, this cutoff was achieved by 7 of 20 PSRE who were observed in the intervention condition in this study. A systematic review and meta-analysis on adjunctive antiseizure medications vs placebo found a weighted pooled risk difference of 21% for reaching the aforementioned cutoff.^29^ Another systematic review and meta-analysis reported an odds ratio of 2.27 in their systematic review and meta-analysis on the efficacy of vagus nerve stimulation vs placebo.^30^ While differences in study design and follow-up duration complicate a direct comparison of effect sizes between these studies and the current study, the magnitude of the effect on seizure frequency observed here is remarkable considering the uncertainty about the mechanism of action of a seizure dog to affect seizure frequency. The potential for a seizure dog to reduce seizures may be explained by the bidirectional pathophysiologic relationship between stress and epilepsy.^6,31,32^ That is, while the role of stress in the causal pathway of seizures is complex and incompletely understood, previous studies on cognitive and behavioral interventions focused on stress reduction have demonstrated improvements in seizure frequency.^33,34^

Patient-reported seizure frequency is commonly used in clinical studies evaluating epilepsy interventions. However, management of epilepsy is not only about controlling seizures but also about enhancing quality of life. Particularly among PSRE, for whom treatments have repeatedly failed to achieve seizure freedom, improving self-management and self-efficacy and appropriately managing anxiety symptoms and comorbidities is of fundamental importance for their quality of life. Therefore, to provide insight into the full potential benefits of seizure dog partnership, it is crucial to consider secondary outcomes. Besides a reduction in seizure frequency, this study showed an improvement on different quality-of-life outcomes. An improvement in quality of life after partnering with a trained seizure dog is consistent with findings from 2 self-reported survey studies.^11,12^ Among the several quality-of-life instruments included in this study, the intervention effect was most evident for the EQ-5D-5L, which is an established instrument for obtaining generic HRQoL values for inclusion in cost-effectiveness analyses.^35,36^ With a 2.5% reduction in utility decrement for each consecutive 28-day period with the intervention, the mean utility score is expected to increase from 0.674 to 0.764 after 1 year of seizure dog partnership. Taking into account the age-adjusted general population utility value of 0.890, the average utility decrement attributable to epilepsy and comorbidities reduces by 41.7% (from 0.216 to 0.126). The results on the EQ VAS, QOLIE-31-P, and ICECAP-A indicate smaller improvements on the respective outcomes. It is relevant to consider that previous studies have been unable to find statistically significant improvements in QOLIE-31-P and EQ-5D scores, even when a clinically relevant reduction in seizure frequency of 50% or 75% was observed.^37-40^ Hence, the impact of changes in seizure frequency on the quality of life of PSRE might not be fully captured by the instruments used in this study. Nevertheless, this study detected a sizeable change in EQ-5D-5L utilities, which could indicate that seizure dogs may affect the HRQoL of PSRE through changes in seizure frequency and through other mechanisms. Analyses on the dimension scores of the quality-of-life instruments showed reductions in (seizure-related) stress and improvements in social function, stability, and achievement. While a regular companion dog might also provide such benefits,^17^ the results from this analysis are expected to reflect the impact of the training of a seizure dog because one-third of participants already had a regular companion dog at the start of the study. Furthermore, observations taken during the first months after partnering with the seizure dog were attributed to the usual care condition due to the assumed delayed intervention effect. Thus, any quality-of-life effects similar to those of an untrained dog are likely captured in observations in the usual care condition.

The study reported a discontinuation rate of 24% (i.e., 6 of 25). In most cases, the decision to discontinue was made by the participant and seizure dog provider jointly, primarily due to changes in participants' health or living situations. A seizure dog trajectory is a time-intensive and cost-intensive intervention, and discontinuation of a partnership can affect both the person with epilepsy and the dog. Hence, one should weigh the reported benefits of seizure dogs against the risk of discontinuation and its consequences.

This study has several limitations. First, with 25 participants of whom 20 were observed in the intervention condition, this study has a limited sample size. Although the sample size calculation indicated these numbers were sufficient to detect changes in seizure frequency of the magnitude observed in an earlier study,^10,18^ the limited sample size does have implications for the ability to detect changes in secondary outcomes and limits the possibilities for subgroup analyses. As a consequence, the assumption that the effects are identical between the pretrained dog trajectory and the team coaching trajectory could not be verified. Furthermore, small sample sizes may raise concerns about generalizability. However, because the total population of PSRE is small, the participants of this study constitute a considerable proportion of the total population in the Netherlands eligible for this intervention. Hence, the study findings are expected to be generalizable to the current target population. A second limitation is that blinding of participants was not possible. The impact on the study results is expected to be limited because in the analyses, the start of the intervention effect was defined at a later time point than the partnering with the dog, and this delay period was unknown to participants. A third limitation is that the study relied on self-reported seizure frequency data. While self-reported seizure diaries are a common instrument for collecting seizure frequency data in clinical and research settings, the quality of such data depends on accurate recognition and recording of seizures by the person with epilepsy. This can be challenging, especially for seizure types that occur at high frequencies or are nondisabling such as absence seizures or myoclonic jerks. Sensitivity analyses showed that excluding these seizure types did not result in meaningful changes to the effect size. Fourth, the stepped-wedge study design complicates the estimation of the intervention effect at fixed time points of follow-up as the number of participants decreases with increasing time with the intervention. That is, data for 20 participants were available for the time point 12 weeks (3 periods) with the intervention, while for 1 year (13 periods) with the intervention, data were available for 13 participants. Because no indication of a stabilization of the intervention effect was observed in the data, more participants or a longer follow-up would be required for determining the point in time at which the intervention has reached its full potential. Furthermore, follow-up after discontinuation is required to understand the impact of ending the seizure dog partnership on the outcomes reported here. Last, the COVID-19 pandemic coincided with part of the data collection, which may have affected the training and coaching process and the outcomes measured in this study. An additional survey was administered during a lockdown period (May 2020) to gain insight into the potential influence of the pandemic on the outcomes of this study. These data showed no clear impact of the pandemic on seizure frequency and resource use. This is in line with a study conducted in the same period in the United States, which indicated that most people with epilepsy reported no change in seizure frequency during the pandemic.^41^ Moreover, because PSRE switched to the intervention condition at different points in time, any impact of the COVID-19 on study outcomes would have been present in both study conditions and, consequently, have a limited impact on the observed effects of the intervention.

This study represents the most comprehensive and scientifically rigorous examination of the impact of seizure dogs on seizure frequency, seizure-free days, seizure severity, HRQoL, and well-being in PSRE to date. This research showed improvements across all outcome measures except for seizure severity over time with the intervention. Improvements in seizure frequency and generic HRQoL were most sizeable. The high discontinuation rate suggests that seizure dogs may not be suitable for all PSRE, and the prevention and consequences of discontinuation require further study.

## Appendix 1 Authors

**Table:**

Name	Location	Contribution
Valérie van Hezik-Wester, MSc	Erasmus School of Health Policy & Management (ESHPM), Institute for Medical Technology Assessment (iMTA), Erasmus Centre for Health Economics Rotterdam (EsCHER), Erasmus University Rotterdam, Rotterdam, The Netherlands	Drafting/revision of the article for content, including medical writing for content; major role in the acquisition of data; study concept or design; and analysis or interpretation of data
Saskia de Groot, PhD	Erasmus School of Health Policy & Management (ESHPM), Institute for Medical Technology Assessment (iMTA), Erasmus Centre for Health Economics Rotterdam (EsCHER), Erasmus University Rotterdam, Rotterdam, The Netherlands	Drafting/revision of the article for content, including medical writing for content; study concept or design; and analysis or interpretation of data
Tim Kanters, PhD	Erasmus School of Health Policy & Management (ESHPM), Institute for Medical Technology Assessment (iMTA), Erasmus Centre for Health Economics Rotterdam (EsCHER), Erasmus University Rotterdam, Rotterdam, The Netherlands	Drafting/revision of the article for content, including medical writing for content; analysis or interpretation of data
Louis Wagner, MD	Academic Center for Epileptology Kempenhaeghe, Heeze, The Netherlands	Drafting/revision of the article for content, including medical writing for content; study concept or design; and analysis or interpretation of data
Jacqueline Ardesch, MD	Stichting Epilepsie Instellingen Nederland (SEIN), Heemstede, The Netherlands	Drafting/revision of the article for content, including medical writing for content; analysis or interpretation of data
Werner Brouwer, PhD	Erasmus School of Health Policy & Management (ESHPM), Erasmus Centre for Health Economics Rotterdam (EsCHER), and Erasmus University Rotterdam, Rotterdam, The Netherlands	Drafting/revision of the article for content, including medical writing for content; analysis or interpretation of data
Isaac Corro Ramos, PhD	Erasmus School of Health Policy & Management (ESHPM), Institute for Medical Technology Assessment (iMTA), Erasmus Centre for Health Economics Rotterdam (EsCHER), and Erasmus University Rotterdam, Rotterdam, The Netherlands	Drafting/revision of the article for content, including medical writing for content; study concept or design; and analysis or interpretation of data
Saskia le Cessie, PhD	Leiden University Medical Center, Leiden, The Netherlands	Drafting/revision of the article for content, including medical writing for content; study concept or design; and analysis or interpretation of data
Matthijs Versteegh, PhD	Huygens & Versteegh, Zwijndrecht; Erasmus School of Health Policy & Management (ESHPM), Institute for Medical Technology Assessment (iMTA), Erasmus Centre for Health Economics Rotterdam (EsCHER), and Erasmus University Rotterdam, Rotterdam, The Netherlands	Drafting/revision of the article for content, including medical writing for content; study concept or design; and analysis or interpretation of data
Job van Exel, PhD	Erasmus School of Health Policy & Management (ESHPM), Erasmus Centre for Health Economics Rotterdam (EsCHER), and Erasmus University Rotterdam, Rotterdam, The Netherlands	Drafting/revision of the article for content, including medical writing for content; study concept or design; and analysis or interpretation of data

## Appendix 2 Coinvestigators

**Table:**

Name	Location	Role	Contribution
Marie-Jose Enders-Slegers, PhD	Open University, Institute for Anthrozoology, Ammerzoden, the Netherlands	Professor em. in Anthrozoology	Conception and design of the study, sharing knowledge on (the assessment of) the benefits and challenges of service animals and the human-animal bond
Laura M'Rabet, PhD	EpilepsieNL, Houten, the Netherlands	Board member knowledge & innovation	Interpretation of data, sharing knowledge on and experience with the impact of epilepsy on patient and family
Helma Verhoeven-Jacobs	None	Patient representative	Interpretation of data, sharing knowledge on and experience with the impact of epilepsy on patient and family and sharing experiences with the placement of a seizure dog

## Glossary

EPISODE: EPIlepsy SuppOrt Dog Evaluation
GLMM: generalized linear mixed modeling
HRQoL: health-related quality of life
PSRE: people living with severe medically refractory epilepsy
RCTs: randomized controlled trials
